# School Hygiene

**Published:** 1905-01-07

**Authors:** 


### SCHOOL HYGIENE.

THE Council of the Royal Sanitary Institute has made the important announcement that a conference on School Hygiene, under the auspices of the Institute, and as a preparation for the International Conference upon the same subject to be held in London in 1907, will be held in the Great Hall of the University of London from the 7th to the 10th of February next. The conference will be under the presidency of Sir Arthur W. Rücker, M.A., D.Sc., LL.D., F.R.S., who will open the proceedings by an address on February 7th at 8 P.M., the chair being taken for the occasion by the President of the Institute, the Duke of Northumberland. Meetings will be held on the mornings and afternoons of the subsequent days, and Sir Lauder Brunton, Lord Reay, Sir William Anson, Sir James Crichton Browne, Sir William Collins, and the Bishop of Hereford, are announced as chairmen. A discussion on " Physical and Mental Development during School Life " will be opened by Miss A. J. Cooper, of Oxford ; on " Physical Inspection " by Dr. Chalmers, of Glasgow ; on " Building and Equipment (of Schools) " by Sir Aston Webb ; on " Sanitary Inspection (of Schools) " by Dr. H. Leslie Mackenzie, of the Scottish Local Government Board ; on the " Training (in Hygiene) of Teachers," by Professor C. S. Sherington ; and on the " Training (also in Hygiene) of Scholars," by Professor Findlay. For each of these discussions the names of several speakers are announced, among whom Dr. Clement Dukes, Mrs. Woodhouse, of the Clapham High School, Dr. Newsholme, and Miss Alice Ravenhill, may be specially mentioned ; but opportunities of addressing the meetings will be afforded also to independent visitors. An exhibition of school furniture and appliances will be open during the conference, and luncheon and light refreshments will be obtainable in the building. Particulars and tickets of admission, which cost 10s. 6d. for persons not members of the institute, may be obtained at the offices of the institute, 72 Margaret Street, W.

